# Detection of repeat expansions in large next generation DNA and RNA sequencing data without alignment

**DOI:** 10.1038/s41598-022-17267-z

**Published:** 2022-07-30

**Authors:** L. G. Fearnley, M. F. Bennett, M. Bahlo

**Affiliations:** 1 Population Health and Immunity Division, The Walter and Eliza Hall Institute of Medical Research, 1G Royal Parade, Parkville, VIC 3052 Australia; 2 Department of Medical Biology, The University of Melbourne, 1G Royal Parade, Parkville, VIC 3052 Australia; 3 Epilepsy Research Centre, Department of Medicine, The University of Melbourne, Austin Health, 245 Burgundy Street, Heidelberg, VIC 3084 Australia

**Keywords:** Computational biology and bioinformatics, Data processing, Genome informatics, Software, Genetic variation, Medical genetics

## Abstract

Bioinformatic methods for detecting short tandem repeat expansions in short-read sequencing have identified new repeat expansions in humans, but require alignment information to identify repetitive motif enrichment at genomic locations. We present superSTR, an ultrafast method that does not require alignment. superSTR is used to process whole-genome and whole-exome sequencing data, and perform the first STR analysis of the UK Biobank, efficiently screening and identifying known and potential disease-associated STRs in the exomes of 49,953 biobank participants. We demonstrate the first bioinformatic screening of RNA sequencing data to detect repeat expansions in humans and mouse models of ataxia and dystrophy.

## Introduction

Short tandem repeats (STRs) are nucleotide sequences comprised of repetitive motifs 1–6 nucleotides (nt) in length found within the genomes of all living organisms. They number in the hundreds of thousands and make up 3–4% of a healthy human genome^1^, playing important roles in an array of biological functions^1–3^. Individual tandem repeats may vary in length and be subject to distinct selective pressures^4,5^. STR length is highly variable, and this has been attributed to events including strand-slippage during replication^6^, retrotransposition^7^, unequal crossing over in meiosis^8^, and issues in DNA repair^9^. STR length variation is captured but not routinely evaluated in short-read whole-genome sequencing data, even though bioinformatic methods exist to extract this length information.

This STR length variation, particularly expansions of STRs (repeat expansions, REs), is associated with many phenomena and phenotypes. REs are known to be causally implicated in or associated with a wide range of human diseases. Somatic REs are a hallmark of hereditary non-polyposis colorectal cancers^10^. Germline REs play causal roles in over fifty neurogenetic and movement disorders, including Huntington’s disease, certain epilepsies, myotonic dystrophies, and many ataxias^4,11–13^. The presence of germline expansions associated with neurodegenerative disorders also appears to reduce overall risk of other diseases, including certain cancers^14^. Taken as a whole, these phenomena suggest complex, evolutionarily conserved mechanisms of action worthy of study for their role in human disease and biology.

Sites harbouring STRs within the genome (STR loci) have been identified and catalogued in genomic sequences using a set of well-established bioinformatic approaches^15,16^. Investigation of STRs in individuals, particularly in the clinical context, are performed using laboratory-based methods such as repeat-primed polymerase chain reactions and Southern blotting targeting specific individual motifs at specific genomic loci^17^. These methods are unsuitable for screening of multiple repeats due to their low throughput. The development of long-read sequencing (LRS) has recently been shown to offer immense utility in analysis of STR loci^18^. LRS is particularly useful in uncovering the architecture and sizing of large and complex REs such as those associated with cerebellar ataxia with neuropathy and bilateral vestibular areflexia syndrome (CANVAS)^19^ or benign adult familial myoclonic epilepsy (BAFME)^20^, but the relative expense and high volumes of data associated with obtaining equivalent sequencing depth across the genome offer significant barriers to its use in large-scale studies.

Short-read next-generation sequencing (NGS) based detection of REs via whole-exome and whole-genome sequencing (WES and WGS, respectively) represents the bulk of sequencing data currently available. Analysis of expansion of repetitive elements in NGS data remains uncommon outside of research settings. A set of bioinformatic methods for detection of REs exist but are dependent upon alignment of repetitive regions to a reference. The first generation of bioinformatic RE detection methods for NGS data are reliant on catalogues of known, predefined STR loci identified in the reference genome^21–28^. These methods estimate the size of a putative RE at each potential STR locus, and if able to, proceed to test for statistical significance by case–control or outlier analysis^23,24^. Recent de novo methods perform RE discovery within alignments without prior knowledge of STR loci. These methods search for patterns of poor alignment to the reference genome characteristic of REs. A completely de novo strategy was used in discovery of the causal RE in CANVAS, a large RE not present in the reference genome^29^. Initial detection of this expansion involved identifying candidate genomic regions via linkage analysis^30^, followed by visual inspection of aligned reads in these areas. Such manual approaches are limited by the labour-intensiveness of the process and the number of samples needed for linkage analysis. The ExpansionHunter Denovo (EHDN) method automates de novo STR discovery by implementing heuristics on the mapping quality of alignments of reads to a reference^31^. EHDN was also used in the detection of CANVAS-associated REs^32^. STRetch is another de novo STR discovery method based on realignment of reads to a reference containing STR decoys, a computationally costly step that is complicated by the combinatoric nature of the number of STR contigs generated as decoys as the motif length analysed increases^23^.

Discovery of REs in all such methods relies entirely on information found in alignment to a reference genome. Important and extensive work on ensuring harmonization of alignment and variant calling methods across cohorts and sequencing centres has been implemented in genome and exome aggregation projects including gnomAD and the NCI Genomic Data Commons^33–35^. Such methods have focused on substitution, insertion and deletion, and structural variation, but have not considered harmonization or optimisation for calling of RE status or size. Consistency in the choice of reference genome is also critical, both across reference builds (i.e. GRCh37 vs GRCh38) and within builds where variations on inclusion of alternative contigs and decoy sequences may impact calls. The recent release of a draft complete human genome generated by long-read sequencing^36^ is likely to result in the incorporation of low-complexity regions, including highly-repetitive centromeric and telomeric sequence, into the reference sequence. These changes and sources of variation in alignment have effects which become significant when considering the analysis of data at scale and across individuals and cohorts.

An alternative approach is to simply test all reads for repetition without alignment. Such an approach has been implemented in the TRhist utility, and may be implemented using alternative methods for processing of sequences in FASTA format, such as mreps^37,38^. Both approaches use efficient algorithms based on string decompositions into unique, minimal representations, followed by steps that find maximal periodicities in the decomposed string^39,40^. TRhist was used in the detection of REs associated with familial adult myoclonic epilepsy^20^, neuronal intranuclear inclusion disease, and oculopharyngodistal myopathy^41^. These tools are conceptually similar to the previously published tools used in assembled genomes (Tandem Repeat Finder, tantan, RepeatMasker^15,16,42^) but differ fundamentally when applied in this fashion, in that they process many millions of short genomic sequences rather than small numbers of much longer genomic scaffolds. Sequential processing of all reads causes extremely long processing times when analysing data from NGS experiments with moderate target depth, and no method exists for comparison of groups of samples using either repeat expansion detector.

We present superSTR, the first method demonstrated to rapidly identify REs in population-scale cohorts without alignment in NGS experiments. superSTR uses a fast, compression-based estimator of the information complexity of individual reads to select and process only reads likely to harbour repeat expansions for processing using the linear-time maximal repetition detection algorithm^40^ used in mreps^38^ in individual samples within a cohort, then compares these samples to identify motifs that are associated with diseases or phenotypes of interest. Expansions of motifs and samples identified by superSTR may then be followed up with other methods. We demonstrate superSTR’s ability to identify samples and screen for motifs with REs in raw sequencing data from short-read WGS experiments, in biobank-scale analysis of WES, and for the first time in direct interrogation of repeat sequences in RNA-seq. The demonstration of superSTR on the UK Biobank identifies both known disease signals as well as novel signals which are poised for replication studies in disease specific cohorts.

## Results

### superSTR efficiently and accurately identifies reads and samples containing repetitive sequence

**Figure 1:**
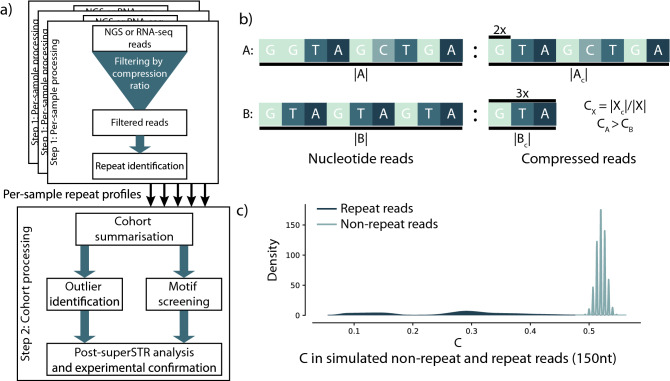
An overview of superSTR, its compression heuristic, and the heuristic’s performance in simulated reads. (**a**) superSTR analysis involves a per-sample processing step where repeats are identified and a cohort-level analysis where samples are analysed, ultimately leading to post-superSTR analysis or experimental confirmation of findings. (**b**) superSTR relies on relative compressibility to distinguish between repeat containing reads. Compression with zlib involves removal of duplication. Read A (which does not contain significant repetition) will compress less than read B (which does), and the ratio of compressed size to uncompressed size will be greater for A than B. B) Distribution of C compression ratios in 150nt pseudorandom reads and repeat-containing reads drawn from a distribution where nucleotides are equiprobable and no errors are present. A more complete characterization across different distributions, read lengths and error rates is contained in Supplementary Figs. S1–S5.

An overview of superSTR is provided in Fig. 1. superSTR efficiently identifies reads containing repetitive genomic sequence for processing by estimating the information content of each read using compression^43,44^. It relies on repetitive sequence being more compressible than non-repetitive sequence^44^. We compute a compression ratio for each read in a NGS experiment, *C,* using the highly optimised zlib compression library^43^. A threshold imposed on this ratio is used to identify lower-complexity reads as demonstrated in simulated sequence data (Fig. 1c), and in simulated reads from experiments targeting repeat expansions generated by computational insertion of STRs into non-repetitive genomic sequence sampled from the human genome (“Supplementary Materials”).

A classifier using *C* to identify repeat-containing reads performs near-perfectly in simulations of both various types of repeat-containing reads and of sequencing experiments of genomic loci containing repeat expansion at read lengths greater than or equal to 75 nucleotides in length (“Supplementary Information”). *C* performance degrades, fewer reads are identified as non-informative, and the classifier becomes less specific as read lengths decrease below 75nt (“Supplementary Information”, Supplementary Fig. S2). We further tested performance of *C* with different sequencing error rates and repeat impurities and observed similarly high performance (Fig. 1c, Supplementary Figs. S3–S8). We provide a set of optimal threshold values for *C* for the distributions and read lengths from these simulations (Supplementary Table 2).

#### Detecting repetitive element outliers in short-read sequencing of individuals with known repeat expansions

We assayed 150nt paired-read whole-genome sequencing data from the Illumina Polaris dataset of 270 individuals. The Diversity cohort contains 150 samples of unascertained RE status, and the Repeat Expansion (RE) cohort contains 120 samples with RE lengths experimentally validated by the Coriell Biobank and the laboratories^26^.

**Figure 2:**
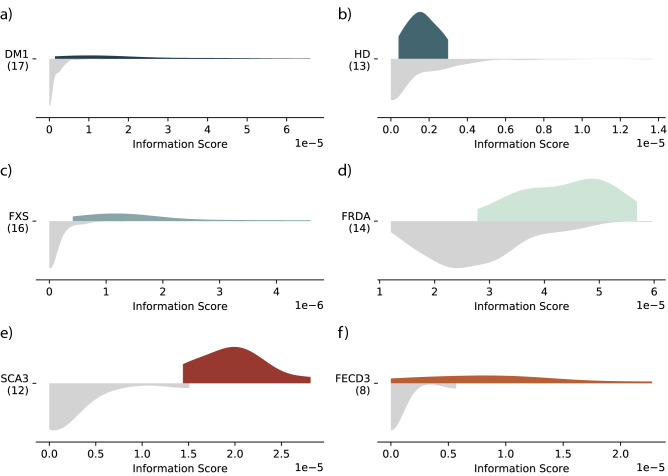
superSTR analysis of WGS and RNA-seq RE data. The distribution of information scores in controls is shown in grey (lower part of each graphic) and affected individuals in color (upper part of each graphic). A right shift in the distribution or the presence of a tail indicates an increased quantity of repeats of that motif in the sequencing data. (**a**–**d**) show comparison of disease groups within the Illumina RE cohort to the Illumina Diversity cohort. (**e**, **f**) show RNA-seq analysis. (**a**) AGC profile of DM1-bearing individuals with long-tailed distribution characteristic of relatively large RE; (**b**) AGC profile of HD-bearing individuals with a much shorter RE; (**c**) CCG profile of FXS individuals; (**d**) AAG profile of FRDA individuals. (**e**) RNA-seq AGC profile of peripheral blood mononuclear cells from 12 individuals with SCA3 RE against 12 matched non-SCA3 controls. (**f**) RNA-seq AGC profile of from eight patients with confirmed FECD3 expansions and six controls without FECD (of any type).

We detected 28 unique motifs with corrected *p*-values (*p*) < 0.05 in at least one disease group of individuals with RE in the Polaris RE cohort (Supplementary Data S1). We observed significant differences in AGC motif median difference in myotonic dystrophy, type 1 (DM1) (*p* = 4.1 × 10^−3^, Fig. 2a) and in Huntington’s disease (HD), which is associated with a smaller CAG expansion (*p* = 3.8 × 10^−2^, Fig. 2b). Shifts in the AAG distribution were significant across the group of individuals with Friedreich’s ataxia (FRDA) and those identified as FRDA carriers (*p* = 4.1 × 10^−3^ and *p* = 1.4 × 10^−2^ respectively, Fig. 2d). We observed significant shifts in the CCG information score in individuals with Fragile X Syndrome (FXS) and again in individuals with premutation-length FXS alleles (*p* = 4.1 × 10^−3^ for both groups, Fig. 2c). Pathogenic AGC, AAG and CCG motifs were only significantly expanded in their associated disease groups. Among the 25 remaining motifs we note that the hexamer AGAGGG significantly differed in all seven groups; the next most common motif was AGGG, present in five (DM1, FRDA, HD, FXS Premutation and FXS Normal). Many of the repeats detected (including AGAGGG, AAGG, AGG, and AAGGG) have been associated with regions predicted to form G-quadruplex structures^45^, which play complex roles in neurodegenerative disorders^46^.

All 17 DM1 individuals in the Polaris cohort were called as outliers for the AGC motif using the bootstrapped outlier detection method, compared to nine called by ExpansionHunter Denovo’s (EHDN) motif analysis method. The DM1 samples identified by superSTR that were not identified by EHDN in analysis ranged from 65 repeats through to 500 repeats in size; the smallest size repeat identified as an outlier by EHDN was 500 repeats. Seven of 14 FRDA cases were called as AAG motif outliers, along with one carrier and a single individual who is likely a FRDA expansion carrier. 13 of 16 FXS cases were called as outliers for the CCG motif, along with 4 FXS premutation individuals, a non-affected relative of a FXS case, and a complex FXS case (NA07063). Full descriptions of outlier calls are contained in Supplementary Data S7.

We did not identify individuals with the relatively smaller Huntington’s disease AGC expansion as outliers using either superSTR or the EHDN motif method. Ranking samples by AGC information score revealed several samples within the Diversity cohort with intermediate AGC scores, but no known AGC pathogenic repeat expansions. We performed follow-up EHDN locus analysis of these individuals, detecting CAG expansions in *TCF4* (associated with Fuchs’ endothelial corneal dystrophy type 3 (FECD3)), an intronic CAG expansion in the *CA10* gene, and an apparent heterozygous expansion at the locus associated with spinocerebellar ataxia type 1 (SCA1) in *ATXN1*. The finding of a SCA1 expansion was not supported by confirmatory targeted-locus analysis using ExpansionHunter^26^.

### Motif screening in the UK Biobank

We obtained and analysed 75nt paired-read WES data for 49,953 individuals with GP and hospital-level clinical records mapped to ICD10 coding from the UK Biobank’s FE WES dataset^47^ as described in the Methods. We performed superSTR analysis on all individuals, and analysed differences in the information scores across all tri-, tetra- and pentamer motifs (a total of 145 motifs) within four-character UK Biobank ICD10 codes corresponding to diseases of the nervous system (Chapter VI, G00–G99) and diseases of the eye and adnexa (Chapter VII, H00–H59) between individuals with each diagnosis and the rest of the Biobank. *p*-values were corrected within each ICD code using Benjamini–Hochberg correction^48^ across the motifs detected in the set of individuals with each ICD10 code. Motifs were reported against an ICD code if they met a significance threshold of 0.05 after multiple-testing correction. Each sample with an ICD code identified as having a potentially significant associated motif was then processed with ExpansionHunter Denovo to localize expansions of the motif within that sample.

We identified 46 unique motifs achieving statistical significance. These were comprised of six trimers, 10 tetramers, and 30 pentamers in 60 disorders (Table 1, Supplementary Data S2). The AGC motif information score distribution was significantly greater in individuals with myotonic disorders (G711, *n* = 13, *p* = 3.4 × 10^−3^) than those without, consistent with presence of DM1. Significant differences in AGC scores were also observed for individuals with hereditary corneal dystrophies (*n* = 93, *p* = 6.3 × 10^−3^), consistent with presence of FECD3. Follow-up analysis with EHDN localized these putative expansions to the expected *DMPK* and *TCF4* genes for 77% and 67% of these samples respectively, along with several other loci (44 additional genes for G711, and 131 for H185). Among the genes to which EHDN mapped CAG repeats in individuals diagnosed with hereditary corneal dystrophies (H185) were genes with non-RE mutations linked to ocular disorders and dystrophies, including *AGBL1* (*n* = 10, causal for FECD type 8^49^), *PDK3* (*n* = 23, Charcot-Marie-Tooth disease, type 6, which involves development of optic atrophy among other symptoms^50^), *POLG* (*n* = 2, progressive external ophthalmoplegia^51^), and *RAB28* (*n* = 1, previously associated with cone-rod dystrophies^52^).

**Table 1:** superSTR analysis of UK Biobank data—significant trimers in motif screening of ICD10 codes and post-screening localization of reads in samples and codes identified by superSTR by EHDN.

ICD	ICD description	# samples in cohort with ICD	Motif	MW statistic	FDR-corrected p-value	# samples with EHDN paired IRRs	EHDN localised genes (n, mean estimated het. allele lengths)
H185	Hereditary corneal dystrophies	93	AGC	3113774	0.0064	88	TCF4 (63, 351.0), AGBL1 (10, 78.4), PDK3 (23, 99.95652173913044), POLG (2, 64.0), RAB28 (1, 74.0) & 127 other genes
G911	Obstructive hydrocephalus	14	AAC	430504	0.035	1	CHID1 (1, 97.0), TNFRSF19 (1, 62.0)
G711	Myotonic disorders	13	AGC	530258	0.0034	13	DMPK (10, 1736.7), 44 additional genes (including MLTT3, TCF4, CA10, TBP)
G568	Other mononeuropathies of upper limb	15	AGG	5332345	0.038	0	DCAF8L2 (7, 86.4), FGFR4 (2, 68.5), CHD3 (1, 61.0), EPHB3 (1, 61.0), GDNF (1, 45.0), WIZ (1, 117.0), NAA38 (1, 61.0), ERICH6 (1, 48.0)
G439	Migraine, unspecified	2352	ACG	56157179	0.036	5	CASZ1 (3, 62.0)
H654	Other chronic nonsuppurative otitis media	95	ACG	2414382	0.023	1	No localisation
G440	Cluster headache syndrome	2082	CCG	51810209	0.045	113	FMR1 (1044, 94), AR (532,74) and 113 additional genes
G440	Cluster headache syndrome	2082	ACG	50023184	0.020	5	CASZ1 (3, 62.0)
H269	Cataract, unspecified	2680	ATC	64490674	0.017	27	CENPP (10, 58.8), ASPN (10, 58.8) and 41 other genes not previously linked to cataract disorders
G048	Other encephalitis, myelitis and encephalomyelitis	17	ACG	448775	0.038	1	No localisation
H210	Hyphaema	29	ATC	849421	0.033	1	ABCA8 (1, 168)

Repeat sizes are as reported by EHDN, and are listed for qualitative purposes only due to high uncertainty resulting from low coverage and numbers of supporting reads in some areas of the WES data. A single individual may have expansions of the same motif at multiple loci. IRRs refer to paired reads detected by EHDN where both reads are repetitive, with the same motif, and > 90% of the reads is the specified motif. Highlighted genes have associations with related disorders. An extended version of the above table with complete gene lists incorporating data from tetramers and pentamers is included in Supplementary Data S2.

We observed a statistically significant difference in the AGG motif for mononeuropathies of the upper limb (G568, *n* = 15, *p* = 3.8 × 10^−2^). Follow-up analysis of those individuals with EHDN localised AGG expansions to *DCAF8L2* in 7 participants, *FGFR2* in 2 participants, and *EPHB3, ERICH6*, *CHD3*, *GDNF* and *WIZ* in 1 participant each. Five of these genes have links to neuropathies. *DCAF8* (a paralog of *DCAF8L2*) has been associated with giant axonal neuropathy 2^53^, *FGFR2* disruption leads to axonal neuropathy^54^, and *GDNF* reduces symptoms of neuropathy in mouse models^55^. The mouse ortholog of *CHD3* participates in the nucleosome remodeling and deacetylase chromatin remodeling complex (NuRD), a complex required for peripheral nerve myelination which causes neuropathy in mouse models when disrupted^56^. *EphB3*, another murine ortholog of a screen result, is involved in adult axonal plasticity and recovery from injury^57^.

We detected statistically significant differences in CCG motif information score for cluster headache syndrome sufferers (G440, *n* = 2082, *p* = 4.5 × 10^−2^). EHDN localized those reads to the Fragile X-associated *FMR1* locus for 50% of these individuals. FXS premutation length expansions have previously been associated with migraine headache in family studies of fragile-X associated tremor ataxia syndrome and in relatives of individuals diagnosed with FXS^58^.

The ACG motif is an exceptionally uncommon microsatellite in the human genome, appearing in only 16 genomic locations across the genome, of which only three appear in exons^59^. We were able to detect several highly significant differences in ACG distributions which associated with particular phenotypes. A group of 11 individuals were diagnosed as having both migraine (G439, *n* = 2352, *p* = 3.6 × 10^−2^) and cluster headache syndrome (G440, *n* = 2082, *p* = 1.9 × 10^−2^). Analysis with EHDN localized ACG repeats in the exomes of two of these individuals to the vicinity of exon 21 of *CASZ1*, which appears to be the main driver of significance in this group due to the rarity of this motif. *CASZ1* is a zinc finger transcription factor expressed during brain development^60^ in which a single nucleotide polymorphism has previously been associated with migraine by meta-analysis and GWAS with odds ratio [1.06–1.17]^61^.

### Direct detection of repetitive elements in RNA-seq data

#### Spinocerebellar ataxia type 3

SCA3 is caused by heterozygous expansion of CAG in *ATXN3*. Incomplete penetrance is observed above 44 repeats, and pathogenicity occurs when > 52 repeats are present^62^. We analysed RNA-seq data of peripheral blood mononuclear cells (PBMCs) from 12 individuals with SCA3 and 12 age- and gender-matched control samples^63^. The AGC motif information score distribution was significantly greater than the control group (*p* = 2.9 × 10^−3^, Fig. 2e, Supplementary Data S3). Outlier detection identified all ten SCA3 cases as outlier samples against the control group.

#### Fuchs endothelial corneal dystrophy, type 3

FECD3 is a subtype of Fuchs endothelial corneal dystrophy (FECD) associated with an intronic heterozygous CTG expansion in *TCF4*. Presence of more than 40 repeats is associated with significantly increased risk of FECD^64^ but the expansion is incompletely penetrant. We obtained an RNA-seq dataset describing corneal endothelium of 12 eyes from 12 persons diagnosed with FECD undergoing transplantation and six controls of donor eyes unsuitable for transplant^65^. Eight of the FECD samples were confirmed to have the *TCF4* expansion by STR-targeting and triplet-primed PCR, and the remaining four determined to have non-expansion FECD. *TCF4* expression is high in the corneal endothelium, and there are conflicting reports as to whether the presence of the CTG expansion increases expression of the *TCF4* mRNA^66–68^. We identified a significant increase in information score for AGC motifs in individuals with CAG expansions in *TCF4* (*p* = 1.5 × 10^−2^, Fig. 2f, Supplementary Data S4). Outlier detection identified five of eight individuals with *TCF4* expansions as outliers compared to controls.

### Myotonic dystrophy 1 and long CTG repeat cell line experiments

Myotonic dystrophy, type 1 (DM1) is caused by a CTG expansion in the 3’ untranslated region (UTR) of *DMPK*. The pathogenic range is broad, starting at 50 repeats for mildly-affected individuals and can exceed 2,000 repeats in individuals with severe disease. DM1 expansions induce formation of nuclear RNA foci that can be visually detected by fluorescent in situ hybridization (RNA FISH)^69^.

We analysed data from two studies with RNA-seq of cells treated with CUG-degrading dCas9^70,71^. The first study was performed in myotubes derived from induced pluripotent stem cells from a DM1 patient and an unaffected individual^70^. superSTR analysis was unable to detect differences between treatment and non-treatment groups (*p* = 0.31 for the expected AGC motif, Supplementary Data S5). Investigation revealed the complete absence of long AGC sequences in the RNA-seq data despite the confirmation of their presence by RNA FISH. The second study was conducted in skeletal muscle from DM1 model HSA^LR^ mice that express > 200 AGC repeats^71^. superSTR identified AGC as the sole outlier motif (*p* = 1.2 × 10^−3^, Supplementary Data S6) in HSA^LR^ mice treated with non-targeting dCas9 (*n* = 4) compared to the wild-type mice and HSA^LR^ mice treated with UGC-degrading Cas9 (*n* = 16). Outlier detection based on information scores correctly identified all four repeat-bearing HSA^LR^ mice as outliers with no false positives.

### Execution time and computational benchmarking

RE detection methods rely on statistical analysis of their output. These analyses are performed in two ways: (1) case versus control analysis, where test statistics from individuals with possible REs are compared to a set of controls assumed to be free of pathogenic REs, or (2) outlier detection, where a set of samples of unascertained status undergo analysis to detect outliers. The latter study design assumes a heterogeneous cohort where only a small proportion of the cohort harbour a particular disease-causing RE, forming a potential group of outliers. If the outlier group is too large many of the statistical approaches will fail^11,24^.

No statistical analysis methods are provided for TRhist; and as a result we were only able to evaluate its execution time, not its RE detection performance. We benchmarked TRhist, EHDN and superSTR using RE data from the Polaris dataset as described in the “Supplementary Materials”.

In testing on WGS data, superSTR processed an average of 444 million paired reads per individual within 4.5 h (mean 258 min) using one CPU and less than 200 MB of RAM. TRhist execution times exceeded a cut-off at 36 h. EHDN completed analysis with mean execution time of 24 h 53 m and maximum time of 30 h 46 m. EHDN execution times were dominated by the alignment step, which required up to 15 CPUs and 30 GB of RAM at peak demand; EHDN consistently processed prepared alignments within an hour using a single CPU with peak memory demand of 2.5 GB of RAM.

Both superSTR motif screening and EHDN case–control motif analysis identified statistically significant enrichment of the pathogenic ACG motifs in DM1, AAG motifs in FRDA, and CCG motifs in FXS. superSTR, but not EHDN, identified statistically significant enrichment of pathogenic AGC (CAG) motifs across the Huntington's disease (HD) cohort. At the individual sample level, superSTR outlier detection consistently outperformed EHDN motif-level outlier analysis in identifying pathogenic samples. EHDN showed a recall of 0.53 for DM1-affected individuals called as AGC motif outliers compared to superSTR’s recall of 1.0; 0.75 for FXS-affected individuals (outliers for CCG) compared to 0.81, and 0.29 for FRDA-affected individuals (AAG outliers) compared to 0.50. A lack of orthogonal validation across all STR loci in the Polaris data prevents calculation of statistics requiring evidence of negative status, such as precision or F1-score. Neither superSTR nor EHDN identified HD individuals as AGC motif outliers. A key difference between the two methods is that the primary purpose of EHDN is to generate results at the locus level, however superSTR can only be directly compared to EHDN’s motif-based analysis, which it outperforms. Thus, although the methods can be compared, they should be viewed as complementary. A detailed description of outlier calls for each sample is contained within the “Supplementary Information”.

superSTR and TRhist were computationally benchmarked on SCA3 RNA-seq samples described previously with an average library size of 15 million paired reads. EHDN does not support RNA-seq data. superSTR required mean execution time of 22 m using 1 CPU and 5 MB of RAM; TRhist required mean execution time of 4 h 34 m using 1 CPU and 2 GB of RAM. Detail of benchmarking and results is provided in “Supplementary Information” and Supplementary Figs. S9 and S10.

## Discussion

superSTR fundamentally differs from most existing bioinformatic STR detection methods in performing de novo searches for REs that requires neither alignment nor specification of the locations of putative repeats. superSTR furthermore sets up a statistical testing framework for motif screening and outlier detection, and provides an opportunity to detect REs in the many samples held in repositories such as dbGaP by enabling rapid screening and re-screening of such data where computational resources are scarce or where datasets are prohibitively large. These data sources contain immense potential for discovery of both known, but undetected, as well as novel REs, an area of intense research interest.

The computational tool closest in capabilities to superSTR is the similarly alignment-free TRhist. superSTR substantially computationally outperforms that method by tenfold for execution time by exploiting inherent properties of genomic reads. TRhist, a prototype RE analysis tool, unlike superSTR, has no statistical analysis framework to evaluate signals, further preventing screening of biobank style cohorts.

superSTR outperforms the motif-based analysis of the alignment-dependent EHDN. This is likely due to a number of factors, one of which is the ability of superSTR to make use of signal from reads that only contain partial repeats. As a result, superSTR can detect motif-level changes in shorter RE disorders such as Huntington's disease. EHDN's motif strategy analyses only pairs of reads that are mostly comprised of repeats with the same motif, whereas the locus-based analysis makes use of single reads comprised of repeats. Thus EHDN motif analysis cannot be expected to detect expansions shorter than approximately the fragment length (twice the read length plus the insert size). superSTR also significantly outperforms EHDN for motif-based outlier detection due to fundamental differences in how the threshold for calling outliers is estimated. superSTR defaults to using the conservative upper 95% CI estimate of the 95th quantile of the information score by the BCa bootstrap in only control samples with unascertained status. This behaviour is modifiable by the end-user through parameters. EHDN performs case resampling using both cases and controls using an assumption of a 5% outlier fraction. This assumption is violated in the Polaris cohort and may also be violated in other cohorts for particular REs where it routinely generates high rates of false negatives at loci where repeats are relatively common, such as the FECD3-linked *TCF4* locus. An appropriate selection of the outlier fraction is analysis-specific and should be determined by the user based on the rarity of the phenotype studied in the background population. For example, backgrounds containing individuals of unascertained status, as occurs comparing neurological disease cohorts to non-neurological disease cohorts, may require use of the prevalence of the disease in the population studied as outlier fractions; alternatively, more-stringent criteria may be merited when analysing backgrounds using true control samples).

We recommend use of superSTR in a complementary fashion to existing RE detection tools such as EHDN, EH and exSTRa. superSTR can be used to screen large cohorts for multiple phenotypes to identify outlier repeat motifs and enable any necessary realignment and follow-up with EHDN locus-based analysis for specific sets of individuals and phenotypes to genomically locate the source of the motif signal. It is also useful in studying hypotheses about RE disease that are not necessarily dependent on repeat localisation, such as proposed motif-phenotype correlation in DNA^72^, or in direct assay of potentially toxic repeat-containing RNA^73^. superSTR performs best where the repeat expansion is longer than the read length, and on motifs not common in the genome. superSTR can identify sufficiently large repeat expansions present in the allosome (such as the Fragile X repeat located in the FMR1 gene on the X-chromosome) but is not able to directly account for differences due to chromosome number due to the lack of alignment information. Alignment-based methods currently outperform superSTR when analysing allosomal expansions, and provide localisation and in some cases repeat size estimation, but their performance gains are balanced by the substantially increased computational cost associated with alignment and the significant multiple testing burden created in testing multiple loci per motif across large sets of diseases in cohorts such as the UK Biobank.

Our results in the UK Biobank whole exome sequencing motif screening recovers known RE-disease associations, identifying enrichment of pathogenic motifs in individuals with ICD codes for myotonic dystrophies and hereditary corneal dystrophies. They also identify samples with potentially novel REs localising to disease-linked genes where repetition has not been known to play a role. We caution that there are many significant sources of confounding within the UK Biobank, and that further work is needed to interpret the results presented here, especially since many participants have only partial clinical data. The use of ICD10 categories as labels remains challenging when investigating individuals with RE-associated disease where diseases grouped in a single category under an ICD code may have diverse causes. This may explain the weak p-value associated with the findings related to cluster headache. EHDN identifies a large number of participants with CCG expansions relative to the reference in FMR1, but prior reports of associations between FMR1 expansion and headache emphasise the role of repeat length^58^, complicating matters further. There is also a suggestion of structure or underdiagnosis within the UK Biobank—FECD3 prevalence is estimated as 3–11% in Caucasians^74^, yet the prevalence of the ICD code representing *all* hereditary corneal dystrophies in the analysed cohort is 0.19%, despite the older age of participants in the UK Biobank relative to the general population.

We also note that the UK Biobank WES data analysed here has undergone repeated revisions, with three separate releases of data aligned with different pipelines in response to issues identified in the alignment pipelines. superSTR reads only read sequence from the UK Biobank data, and discards alignment information, and our findings are unaffected by changes to these pipelines. Our findings in the UK Biobank require validation in more disease-specific cohorts and should be treated as leads for further investigation.

We demonstrate that superSTR can directly analyse repeats in RNA-seq data without alignment to reference transcriptomes or genomes. This allows the direct interrogation of transcripts for the presence of potentially toxic repeat-containing RNA, which may be missed in whole-exome sequencing due to involvement of RNA expressed from intronic or intergenic regions (e.g. in non-coding RNAs), or in the increased number of intergenic, non-expressed repetition detected in whole-genome sequencing. Prior studies have largely focused on detection of downstream transcriptional signatures of expansion rather than direct detection of expanded sequence itself. There are several potential complications to such analysis. REs contained in intronic and exonic regions will likely show tissue-specific patterns of expression similar to those of the genes in which they are contained. Intergenic REs may not be expressed at all, although recent work shows that transcription initiation at microsatellites is much more widespread than previously thought ^75^. Choice of RNA-seq library preparation method also appears to impact the viability of STR analysis. Ribosomal RNA depletion methods allow the capture of a wider array of features in RNA than poly-A captured RNA^76,77^ and may be necessary to study intronic RNA (e.g. in TCF4 expansions for FECD3). We observe some evidence of this complexity in DM1-derived myotubes, where the repeat is absent despite confirmation of its presence by RNA FISH imaging. These behaviours may require consideration given the emergent use of cell lines in investigating RE disorders and potential treatments. In summary, superSTR substantially broadens the ability to interrogate REs and will be a valuable tool for genomic discovery work.

## Methods

### superSTR—per sample processing

superSTR processes each read in a next-generation sequencing or RNA-seq experiment by compressing each read. Repetitive reads are more compressible than non-repetitive reads (“Supplementary Information”), and this property is used in read filtering, where those reads that meet a read length-dependent threshold on the ratio of compressed to uncompressed sequence are then processed using a computationally expensive repeat characterisation step. This characterisation is then summarised for each sample for each motif with an information content-based score. Each sample’s scores are then passed to a cohort summarisation step, then used in motif screening (case–control) or outlier detection studies in a cohort of samples. An overview of the superSTR method is provided in Fig. 1.

The per-sample sequence processing component of superSTR (Step 1 of Fig. 1A) is implemented as a single-threaded application in the C programming language. Sequencing data is either read through the htslib library (v1.9) or streamed directly into the application. Each read is compressed using zlib^43^, set for the fastest possible compression speed. *C* is computed as the ratio of the size in bytes of the output data to the size in bytes of the input data (“Supplementary Information”). Thresholds on *C* are read-length dependent and are supplied by the user from either pre-computed threshold tables (as used in this work) or user-run simulation. Reads passing this threshold are then analysed using the Kolpakov-Kucherov algorithm^40^ as implemented in a modified version of the mreps software with optimisation and modifications that enable its use in this context^38^. The repeat detection step used a resolution parameter of 1, corresponding to exact repeat detection, without limitations on the repeat or motif length, and no limit was imposed on the number of unique maximal repeats per read in the detection step. Each read identified as containing repetitive elements has their start and end location, motif, length and repeat purity recorded.

### Cohort processing

Cohort processing consists of a series of steps implemented as Python 3.8 scripts involving summarisation of samples into cohort-level descriptions of the motifs in each sample, followed by motif screening and/or outlier detection. Summarisation produces a repeat count vector $v$ for each motif $m$, $v={v}_{1}, {v}_{2}\dots {v}_{j}$, where ${v}_{n}$ is the number of times a repetitive sequence with motif $m$ and length *n* was encountered in the sequencing experiment. This number is normalised by the experiment’s library size, and per-sample and per-motif files are generated.

These summary vectors are then transformed into an information score, $s$, for each motif ${s}_{m}=({\sum }_{i\le l\le j}{v}_{l}\times l)/n$, where *n* is the number of reads in the sequencing experiment and the *i* and *j* values provide a range of motif lengths for consideration. The default setting used in this study for *j* is the read length and *i* is [*j* × 0.75]; they may also be manually set, which permits detection of some shorter REs. superSTR can handle cohorts where samples have been sequenced using mixed read lengths by trimming of reads to a consistent length, a process analogous to downsampling of the experiments with longer reads. Care should be taken in trimming, and the approach is recommended only across samples where reads are similar in length and insert size (e.g. where some reads have length 150 nt and some 151 nt).

Motif screening is performed on labelled samples to determine whether the distribution of information scores is greater in a group of samples relative to a background, as would be expected if a repeat expansion was associated with the label. superSTR implements this with a one-sided Mann–Whitney U test in scipy^78^, with the alternative hypothesis that randomly selected samples of interest have larger information scores than samples from the background. We computed the p-value of test statistics against a sampling distribution computed by permutation testing of the labels of each sample using 10,000 permutations. p-values were calculated with approximation to the exact value^79^, using the arbitrary-precision mpmath library^80^ to perform the required Gauss–Legendre quadrature. P-values were then false discovery rate-corrected using the Benjamini–Hochberg procedure implemented in statsmodels^81^. Motifs were reported as significant if the corrected Mann–Whitney permutation p-value was less than 0.05 after false discovery rate correction.

Outlier detection in superSTR is performed using a one-versus-many strategy. 95% confidence intervals for the 95th percentile of each motif’s information score are estimated in a background or control cohort using the bCa-bootstrap^82^ as implemented in the arch package (v4.19)^83^, and values that exceed the upper 95% confidence interval of the estimate of this percentile are reported as outliers. superSTR also offers users the option to use a percentile bootstrap should the BCa-bootstrap fail (for example, due to low sample sizes).

All genomic and transcriptomic analysis described were conducted as case–control or outlier detection studies across all motifs detected with multiple-testing correction conducted using the Benjamini–Hochberg procedure to control for false-discovery rates.

### Genome sequence data

The Illumina Polaris cohort consists of 270 samples, comprised of 150 publicly available whole-genome sequences of individuals with unknown RE status (European Nucleotide Archive accession PRJEB20654), and a set of 120 whole-genome sequences from the Coriell Biobank with pathogenic REs of varying lengths, representing eight disorders associated with three motifs (European Genome-Phenome Archive accession EGAD00001003562). Pathogenic REs were characterised and validated by the submitting laboratories and Coriell Biobank in each sample. The latter data is available on application as detailed in the European Genome Archive. Samples for both cohorts were prepared with TruSeq PCR-free sample preparation, then sequenced by an Illumina HiSeq X with 150 × 150 nt paired read lengths to a target whole genome coverage depth of 30×. This dataset has been previously used to evaluate performance of RE detection methods^21,24,25,31^. Both superSTR and EHDN (v.0.9.0) were run on all samples in the Illumina Polaris cohort.

Whole-exome sequences of 49,953 individuals with unascertained RE status and available linked primary care records were obtained from the UK Biobank WES FE dataset in CRAM format (Data Fields 23163, 23164). Read sequences were read directly from the UK Biobank-provided CRAM files for each individual by superSTR. These individuals were sequenced with the DT xGen Exome Research Panel v1.0, targeting a total 39 million base pairs of the genome (19,396 genes). Samples were sequenced with 75 × 75 nt paired-end reads on the Illumina NovaSeq 6000 platform to a target 20× coverage depth. Primary care data containing readv2 and readv3 codes were converted to ICD-10 codes using the UK Biobank-supplied tables (UK Biobank Data Coding 19). A permissive mapping strategy was used in conversion where each read code was mapped to all possible ICD10 codes. Codes mapping to nervous system disease (G00–99) and diseases of the eye and adnexa (H00–H59) were tested for association with trimer, tetramer and pentamer motifs using superSTR’s case–control analysis mode. EHDN was then used to process all samples annotated with ICD10 codes meeting a significance threshold of 0.05 for a motif *p*-value in the superSTR case–control analysis.

### Transcriptome sequence data

All transcriptomic data is sourced from published experiments that are publicly accessible within the NCBI Sequence Read Archive.

RNA-seq data for SCA3 was obtained from a study describing the transcriptome of peripheral blood mononuclear cells in 12 SCA3-affected individuals with 12 age- and gender-matched controls from a study of patients at Xiangya Hospital in Changsha, China^63^ (SRA accession SRP168964). Samples were depleted of ribosomal RNA, cDNA synthesised using the TruSeq Stranded Kit, then sequenced on an Illumina HiSeq X Ten instrument with 100nt long reads.

RNA-seq data for FECD3 was obtained from a study conducted at the S. Fyodorov Eye Microsurgery Federal State Institution, Russia^65^ (SRA accession SRP186882). This study describes the transcriptome of the corneal endothelium of twelve individuals with FECD undergoing corneal transplantation, along with six control samples obtained from deceased individuals. Eight of these samples showed expansion in the *TCF4* gene when assayed using a triplet-primed polymerase chain reaction. Samples were depleted of ribosomal RNA and sequenced with 2 × 125 cycles on an Illumina HiSeq 2500 instrument. Mean read lengths of the data deposited in the Sequence Read Archive for this experiment varied among samples from 132 to 124 nt. We trimmed reads in this experiment to a uniform 124nt length prior to processing with superSTR.

RNA-seq data for myotonic dystrophy, type 1 (DM1) was obtained from two studies of clearance of CTG-repeat RNA foci by RNA-targeted CRISPR-dCas9. The first study was conducted in human myoblasts derived from induced pluripotent stem cells that were in turn derived from a patient with DM1 (SRA accession SRP111361)^70^. These cells have a CAG repeat length estimated by multiple orthogonal methods as being between 1793 and 2700 repeats in the *DMPK* gene^84^. This study used Illumina TruSeq PolyA library preparation, and samples were sequenced on an Illumina HiSeq 4000 instrument, with a uniform 99 × 99 nt read length.

RNA-seq data for DM1 in a mouse model was obtained from a study of clearance of CTG-repeat RNA foci in skeletal muscle of HSA^LR^ mouse, which expresses 250 CTG repeats associated with the human skeletal actin promoter, and has 20–25 CTG repeats in the mouse *Dmpk* gene itself (SRA accession SRP266474)^71^. This study used Illumina TruSeq PolyA library preparation, and samples were sequenced on an Illumina HiSeq 4000 instrument. The SRA-deposited RNA-seq data for this experiment also varied in average spot length, with common read lengths in wild-type mice of 150 × 150 nt paired reads and 100 × 100 nt paired reads in the DM1-affected mice. We cropped reads in this experiment to a uniform 100 nt length prior to processing with superSTR. We assigned ‘expanded’ status to runs of samples from untreated HSA^LR^ mice in this experiment (8 runs) and ‘contracted’ status to treated HSA^LR^ mice and all WT mice in the experiment (12 runs).

### Simulation studies of C as a metric for classifying repetitive reads

We evaluated superSTR’s *C* ratio in classifying simulated repeat-containing reads against pseudorandom sequence drawn from four background nucleotide distributions. The uniform distribution, where each nucleotide is equiprobable, and distributions representing the genomic distributions of *Homo sapiens*, the GC-rich bacterium *Streptomyces coelicolor*, and the AT-rich intergenic regions of the protozoan *Plasmodium falciparum*. We randomly selected repeat motifs up to 15 nt with all possible repeat lengths up to the read length (“Supplementary Information”). We tested *C* on common read lengths for experiments deposited in the NCBI Sequence Read Archive, noting that shorter read lengths are generally associated with older data sets.

Additionally, we generated a set of 220,570 simulated NGS experiments targeting simulated repeat expansion loci. A set of non-repetitive sequences, each > 5000 nt in length, was obtained from a UCSC *Homo sapiens* reference in which repetition identified by RepeatMasker and Tandem Repeats Finder had been masked. Simulated STR loci were then generated by inserting repeats into the centre of randomly selected non-repetitive sequences. All known pathogenic STR motifs were represented in this set at their smallest literature-reported pathogenic length in a set of 10 experiments. A further 31,500 loci were generated by repeated, random generation of repeats at different combinations of motif length, total repeat length, and probability of a motif change (“Supplementary Materials”). PCR-free NGS reads on the Illumina HiSeq X Ten instrument were then simulated using the ART read simulator^85^ at common read lengths reported in the Sequence Read Archive. Reads were labelled as being repeat-informative if they contained at least 45 nucleotides of repetitive sequence, and the performance of thresholds imposed on *C* as a classifier were evaluated against this labelling (Supplementary Figs. S6–S8).

### Benchmarking

We benchmarked the computational performance of superSTR for WGS and RNA-seq on FASTQ input against TRhist and ExpansionHunter Denovo 0.9.0 (including the alignment, sorting and indexing steps performed with BWA v0.7.17 and samtools v1.11). Execution time was benchmarked on four WGS and four RNA-seq samples on the Walter and Eliza Hall Institute of Medical Research’s HPC system (Intel Xeon Sandy Bridge E5-2690 v4 CPUs with data stored on a Quantum Xcellis Storage Appliance). Accuracy was benchmarked using the Illumina Polaris dataset, testing disease groups within the Repeat Expansion cohort against the ‘control’ population of the Diversity cohort. Full details of benchmarking are contained in the “Supplementary Information”.

## Supplementary Information

Supplementary Information 1.

Supplementary Information 2.

## Data Availability

superSTR is open-source software, and instructions for its use and installation are available in a GitHub repository at https://github.com/bahlolab/superSTR. RNA-seq data used in this study is publicly accessible through the NCBI Sequence Read Archive under accessions SRP168964, SRP186882, SRP111631, and SRP266474. WGS sequencing for the Illumina Repeat Expansion cohort is available on application to the European Genome-Phenome Archive at accession EGAD00001003562. WGS sequencing for the Ilumina Diversity cohort is accessible at the European Nucleotide Archive under accession PRJEB20654. UK Biobank data is available under field ID 23163 ‘Exome FE CRAM files—initial 50 k release’ on application to the UK Biobank.
